# Nonword repetition in adults who stutter: The effects of stimuli stress and auditory-orthographic cues

**DOI:** 10.1371/journal.pone.0188111

**Published:** 2017-11-29

**Authors:** Geoffrey A. Coalson, Courtney T. Byrd

**Affiliations:** 1 Department of Communication Sciences and Disorders, Louisiana State University, Baton Rouge, Louisiana, United States of America; 2 Department of Communication Sciences and Disorders, The University of Texas at Austin, Austin, Texas, United States of America; Leiden University, NETHERLANDS

## Abstract

**Purpose:**

Adults who stutter (AWS) are less accurate in their immediate repetition of novel phonological sequences compared to adults who do not stutter (AWNS). The present study examined whether manipulation of the following two aspects of traditional nonword repetition tasks unmask distinct weaknesses in phonological working memory in AWS: (1) presentation of stimuli with less-frequent stress patterns, and (2) removal of auditory-orthographic cues immediately prior to response.

**Method:**

Fifty-two participants (26 AWS, 26 AWNS) produced 12 bisyllabic nonwords in the presence of corresponding auditory-orthographic cues (i.e., immediate repetition task), and the absence of auditory-orthographic cues (i.e., short-term recall task). Half of each cohort (13 AWS, 13 AWNS) were exposed to the stimuli with high-frequency trochaic stress, and half (13 AWS, 13 AWNS) were exposed to identical stimuli with lower-frequency iambic stress.

**Results:**

No differences in immediate repetition accuracy for trochaic or iambic nonwords were observed for either group. However, AWS were less accurate when recalling iambic nonwords than trochaic nonwords in the absence of auditory-orthographic cues.

**Conclusions:**

Manipulation of two factors which may minimize phonological demand during standard nonword repetition tasks increased the number of errors in AWS compared to AWNS. These findings suggest greater vulnerability in phonological working memory in AWS, even when producing nonwords as short as two syllables.

## Introduction

Stuttering is a multifactorial disorder. Both motor and linguistic factors contribute to difficulties persons who stutter have producing and maintaining fluent speech (for review of language and stuttering, see [1], [2]; cf. [3]; for review of speech motor control and stuttering, see [4], [5]). Among the linguistic factors identified, there are significant data to suggest a relationship between phonology and stuttering. Phonological encoding differences have been demonstrated in individuals who stutter across the lifespan (e.g., [6], [7], [8], [9], [10] cf., [11], [12]). Phonological disorders are the most frequent concomitant disorder in children with developmental stuttering ([13]; cf. [3]). The phonological representations of children who stutter also appear to be less specified than their fluent peers (e.g., [6], [14], [15], [16], [17]). A growing body of data suggest that phonological encoding may be more vulnerable to increased phonological demand for adults who stutter (AWS) as compared to adults who do not stutter (AWNS) (e.g., [18], [19], [20], [21], [22]).

**Fig 1:**
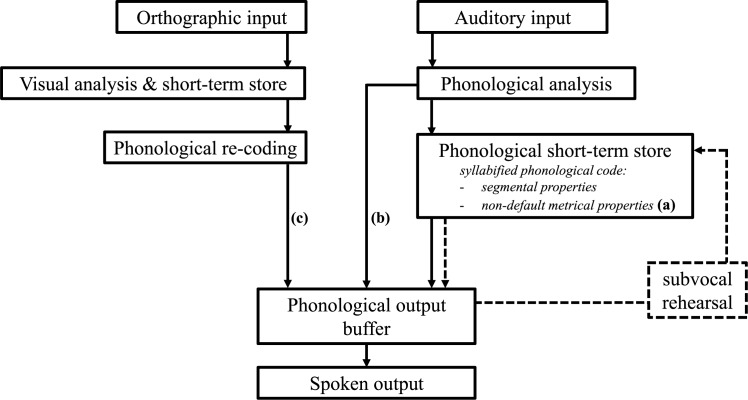
Adapted version of Baddeley’s [25] theoretical model of phonological working memory (i.e., the ‘phonological loop’). (a) Non-dominant stress patterns stored with segmental information in phonological short-term store; (b) Direct access of auditory input to phonological information in short-term store; (c) Direct access of orthographic input to phonological information in output buffer.

Difficulties in phonological encoding in AWS as demands increase would predict that AWS also demonstrate greater difficulty during tasks of *phonological working memory*–such as nonword repetition–wherein verbal accuracy relies heavily on efficient phonological encoding (see [23] and [24] for review of phonological working memory and stuttering). As described by Baddeley’s [25] three-component model, phonological working memory stores and maintains verbal information prior to (or in the absence of) overt production (see Fig 1 for proposed structure of phonological working memory). The ‘phonological short-term store’ provides temporary activation of the speech plan as it is generated by the phonological encoding system. These abstract phonological speech plans–comprised of both segmental and metrical information–remain activated for approximately two seconds prior to decay. Subvocal rehearsal postpones this decay by recruiting speech-motor plans to re-activate information within the phonological store. Subvocal rehearsal becomes less effective, however, as the time required to recruit motor templates exceeds the temporal limits of the phonological store. The limits of subvocal rehearsal in response to phonological demand can be observed during nonword repetition tasks by the well-documented word length effect, wherein repetition accuracy declines as segmental length of the nonword increases.

Nonword repetition tasks have been a valuable tool to assess the limits of phonological processing abilities in persons who stutter due to its reliance on efficient phonological encoding and the ability to manipulate the complexity of nonword stimuli to increase phonological demand. For example, previous studies have found AWS exhibit more robust word-length effects than AWNS. In accordance with multifactorial accounts of stuttering, AWS repetition accuracy is comparable to AWNS when segmental demand is minimal and subvocal rehearsal is unnecessary (i.e., 1- to 4-syllable nonwords [21], [26], [27]). AWS become significantly less accurate than AWNS, however, as segmental length exceeds their temporary store and subvocal rehearsal is required (e.g., 7-syllables [26], [28]; 6-syllables [21]). These data support the notion that phonological working memory–as measured by nonword repetition tasks–is more susceptible to breakdown in AWS than AWNS, provided that the task is sufficiently challenging.

That being said, there are two technical aspects of the nonword repetition tasks used in previous studies that may have inadvertently *decreased* phonological demand and, as a result, may have underestimated phonological working memory weaknesses in AWS. First, stimuli used across the studies were restricted to high-frequency stress patterns. Use of these high-frequency patterns may have minimized metrical processing during encoding, storage, or subvocal rehearsal (see Fig 1A). Second, stimuli were repeated immediately after presentation of either corresponding auditory cues (Fig 1B) or orthographic cues (Fig 1C). Use of either cue may have strengthened activation of the phonological target within the phonological store immediately before the participant’s response. In the present study, we systematically manipulated these two task-related variables to examine whether phonological working memory in AWS–and, by extension, phonological processing–is weaker than previously reported.

### Metrical stress and working memory

To date, phonological working memory in AWS has been examined only with respect to segmental properties, most commonly the number of syllables within a target nonword. Metrical properties, such as syllabic stress, also impose demand on working memory (e.g., [29], [30], [31]). These effects are most evident when the metrical structure of the to-be-remembered items shifts from high-frequency stress patterns (in English [32], trochaic stress or *STRONG*-*weak*) to low-frequency stress patterns (iambic stress, or *weak*-*STRONG*). Although overt errors based on metrical structure are most commonly observed during early development ([33], [34]), metrical stress influences phonological working memory well beyond childhood. For example, Morgan and colleagues [35] found English-speaking adults recalled lists of six monosyllabic nonwords less accurately when presented with iambic stress patterns than trochaic stress patterns (see [36], [37] for additional evidence). These data suggest that, similar to low-frequency segmental properties (e.g., [38], [39]), low-frequency metrical properties may disrupt the retention of phonological targets in typically fluent adults.

Only two investigations of metrical properties and phonological working memory in persons who stutter have been conducted. Hakim and Bernstein Ratner [40] found that children who stutter (*n* = 8) produced a greater amount of phonemic and stress-assignment errors than fluent peers (*n* = 8; 4–8 years old) when repeating 4-syllable nonwords with unfamiliar stress patterns, although differences between groups did not reach significance. Coalson and Byrd [41] reported that, in addition to slower identification of phonemes within iambic nonwords, AWS (*n* = 22) also produced iambic nonwords with more phonemic and stress-assignment errors than AWNS (*n* = 22) during post-trial production. In the same study, there was a significant negative correlation between performance on digit span subtests and post-trial verbal accuracy for AWS, but not AWNS. These findings suggest a unique relationship between low-frequency stress patterns and production accuracy in AWS, and that this relationship may be associated with phonological working memory abilities. However, no studies have directly examined whether metrical stress is a mediating factor in phonological working memory for AWS, as all stimuli used in previous nonword repetition studies in AWS were comprised exclusively ([21], [26], [27], [28]) or predominately [42] of high-frequency stress patterns.

### Auditory-orthographic cues and working memory

Most studies of nonword production in AWS have employed immediate nonword repetition tasks with auditory stimuli presentation ([21], [26], [27], [28], cf. [42], [43]). According to Baddeley ([25], see also [44], [45]), auditory input immediately activates information held within the phonological store (see Fig 1B). Thus, segmental information near the end of longer nonwords is more accurately recalled because of the temporal nature of acoustic cues (i.e., the recency effect). In the case of shorter nonwords, the recency effect may span the entire target and result in near-perfect repetition accuracy in adults (e.g., 2–3 syllables; [46]). This inherent reduction of phonological demand due to auditory cues may also account for the comparable accuracy observed between AWS and AWNS when repeating shorter nonwords (i.e., 1- to 4-syllables; [21], [26], [27]) but poorer accuracy for AWS when repeating longer nonwords (6+ syllables, [21], [26], [28]). Sasisekaran [42] reported a similar pattern of reduced accuracy for AWS (*n* = 9) than AWNS (*n* = 9) when producing 6- and 11-syllable nonwords via orthographic cues, although group differences failed to reach statistical significance (*p* = .07). Differences between AWNS and AWS may emerge for shorter nonwords, however, when no auditory or orthographic cue is provided immediately prior to production. To that end, *short-term recall* tasks may have a distinct advantage over *immediate repetition* tasks when examining the efficiency of phonological working memory in AWS. That is, AWS may produce nonwords less accurately when required to maintain activation via subvocal rehearsal, and without activation from a preceding auditory or orthographic cue.

To date, only Ludlow et al. [43] have used a nonword cueing paradigm that did not require AWS and AWNS to produce nonwords immediately after an auditory or orthographic cue. Participants were trained to associate a 4-syllable nonword, presented in auditory and written form, with a non-linguistic symbol. Participants were then instructed to produce the nonword upon presentation of the symbol alone. AWS exhibited overall poorer accuracy and significantly fewer correct phonemes than AWNS for the final two syllables–the loci most likely to benefit from recency effects during standard nonword repetition tasks. Poorer accuracy for 4-syllable nonwords conflicts with previous studies that report comparable accuracy between AWS and AWNS for 4-syllable nonwords when presented via auditory cues (i.e., [21], [26], [27], [28]), or 6+ syllable nonwords presented via orthographic cues (i.e., [42]). One potential account for this discrepancy is that removal of auditory-orthographic cues by Ludlow et al. [43] minimized auditory-orthographic priming, increased phonological demand, and exposed greater vulnerability of phonological working memory in AWS. Nevertheless, the benefit of auditory-orthographic priming to AWS during tasks of phonological working memory cannot be determined without direct comparison of accuracy in the presence *and* absence of such cues.

### Summary and research questions

The present study examined whether the vulnerability of phonological working memory in AWS is greater than previously reported by manipulating two specific aspects of standard nonword repetition tasks. To examine whether accuracy was influenced by stimuli with high-frequency stress patterns, both groups produced identical nonwords presented with either trochaic stress or iambic stress. To examine whether accuracy was influenced by cueing methods, participants were first required to repeat these nonwords immediately after auditory-orthographic cues, and then recall the same nonwords upon presentation of a visual symbol that was non-auditory and non-orthographic in nature. We predicted that iambic stress would decrease production accuracy in AWS more so than trochaic stress, and that these differences would become more evident in the absence of preceding auditory-orthographic cues. Specifically, we asked the following research questions:

1. Do AWNS and AWS differ in accuracy when repeating trochaic and iambic nonwords immediately after auditory-orthographic cues?
2. Do AWNS and AWS differ in accuracy when recalling trochaic and iambic nonwords in the absence of auditory-orthographic cues?

## Materials and method

### Participants

The current study and consent form documentation was approved by both authors’ universities (Louisiana State University, IRB #3428, and the University of Texas at Austin, IRB #2012-08-0011). Fifty-two participants (26 AWS, 26 AWNS) ranging in age from 18 to 36 years (AWS: *M* = 22.19, *SD* = 3.30; AWNS: *M* = 22.00; *SD* = 4.17) were included in the present study. All participants completed two 90-minute sessions. During the first 90-minute session, general demographic information, language history, and medical history were collected, along with hearing and vision screening, a conversational speech sample, and a series of phonological processing subtests. Participants provided oral and written consent to participate in the study, in accordance with approval of the Institutional Review Board for both universities. To qualify for inclusion, all participants met the following criteria: (a) 18 years or older, (b) no reported or observed neurological, medical, literacy, language or speech concerns (with the exception of stuttering in AWS), (c) no current use of psychotropic medications, (d) passed binaural pure-tone hearing screening [47], (e) passed a vision screening [48], and (f) native-like proficiency in English (*Language History Questionnaire* [49]; *Language History Questionnaire 2*.*0* [50]).

### Phonological processing subtests

Phonological processing subtests were administered to ensure that participants did not present with clinically significant deficits in phonological encoding and/or working memory abilities, and to account for potential subclinical differences in baseline abilities between groups that may affect performance irrespective of stimuli manipulation. Five subtests that measure phoneme segmentation and phonological working memory were completed, including: (a) word segmentation (*Comprehensive Test of Phonological Processing*, CTOPP [51], Subtest XI), (b) nonword segmentation (CTOPP, Subtest XII; *Comprehensive Subtest of Phonological Processing–Second Edition*, CTOPP-2 [52], Subtest VI), (c) nonword repetition (CTOPP, Subtest V; CTOPP-2, Subtest V), (d) forward digit span (*Wechsler Adult Intelligence Scale–Third Edition*, WAIS-3 [53]; CTOPP-2, Subtest VIII), and (e) backward digit span (WAIS-3; CTOPP-2, Subtest VIII). Updated versions of standardized measures were used as each became available during data collection. Performance on word segmentation, nonword segmentation, nonword repetition, forward digit span and backward digit span subtests were converted to *z*-scores to accommodate for differences between measures. There were no significant group differences between AWNS and AWS across subtests of phonological segmentation (word segmentation: *p* = .18; nonword segmentation: *p* = .36), and phonological working memory (nonword repetition: *p* = .85; forward digit span: *p* = .19; backward digit span: *p* = .98).

### Talker group classification

Participants were considered AWS if they met the following criteria: (a) self-identification as a person who stutters with onset prior to 7 years of age, and (b) prior diagnosis of stuttering by a licensed speech-language pathologist. If the participant had not received a prior diagnosis of stuttering, but identified as a person who stutters, AWS status was confirmed by the first author, an ASHA-certified speech language pathologist. Stuttering severity for all participants is provided in Table 1.

**Table 1:** Participant characteristics for adults who do and do not stutter (AWS, AWNS).

P	SSI	Severity	Dx	Self-ID	P	SSI	Severity	Dx	Self-ID
*Trochaic*					*Iambic*				
AWS-1	13	very mild	N	Y	AWS-14	13	very mild	N	Y
AWS-2	18	mild	Y	Y	AWS-15	12	very mild	Y	Y
AWS-3	19	mild	N	Y	AWS-16	13	very mild	Y	Y
AWS-4	27	moderate	Y	Y	AWS-17	10	very mild	Y	Y
AWS-5	10	very mild	N	Y	AWS-18	22	mild	Y	Y
AWS-6	11	very mild	N	Y	AWS-19	14	very mild	Y	Y
AWS-7	10	very mild	N	Y	AWS-20	11	very mild	Y	Y
AWS-8	24	mild	Y	Y	AWS-21	10	very mild	Y	Y
AWS-9	18	mild	Y	Y	AWS-22	23	mild	Y	Y
AWS-10	15	very mild	N	Y	AWS-23	10	very mild	Y	Y
AWS-11	26	moderate	Y	Y	AWS-24	33	severe	Y	Y
AWS-12	16	very mild	N	Y	AWS-25	14	very mild	Y	Y
AWS-13	14	very mild	Y	Y	AWS-26	32	severe	Y	Y
AWNS-1	6	none	N	N	AWNS-14	5	none	N	N
AWNS-2	5	none	N	N	AWNS-15	5	none	N	N
AWNS-3	4	none	N	N	AWNS-16	4	none	N	N
AWNS-4	5	none	N	N	AWNS-17	5	none	N	N
AWNS-5	6	none	N	N	AWNS-18	7	none	N	N
AWNS-6	4	none	N	N	AWNS-19	4	none	N	N
AWNS-7	5	none	N	N	AWNS-20	4	none	N	N
AWNS-8	7	none	N	N	AWNS-21	4	none	N	N
AWNS-9	4	none	N	N	AWNS-22	7	none	N	N
AWNS-10	6	none	N	N	AWNS-23	5	none	N	N
AWNS-11	5	none	N	N	AWNS-24	4	none	N	N
AWNS-12	5	none	N	N	AWNS-25	7	none	N	N
AWNS-13	9	none	N	N	AWNS-26	4	none	N	N

P: participant; SSI: score on *Stuttering Severity Index-4*; Dx: previous diagnosis of stuttering; Self-ID: participant self-identification as an adult who stutters.

### Stuttering severity

Stuttering severity was determined by the first author from video-recorded conversational samples per the scoring procedure of the *Stuttering Severity Index-4* (SSI-4 [54]). Of the 13 AWS in the trochaic condition, seven received a score of “very mild,” four received a score of “mild,” and two received a score of “moderate.” Of the 13 AWS in the iambic condition, nine received a score of “very mild,” two received a score of “mild,” and two received a score of “severe.” None of the AWNS reported prior diagnosis of stuttering, or self-identified as a person who stutters. AWS participants in the trochaic and iambic conditions did not significantly differ in SSI-4 severity classification [χ^2^ (4, *n* = 13) = 2.91, *p* = .574].

Inter-rater reliability of stuttering severity was established by the first author and one graduate research assistant trained in disfluency count analysis based on the SSI-4 severity classification. Sixteen of the 52 participants (i.e., 30%; eight AWNS, eight AWS) were randomly selected to determine inter-rater reliability. Inter-rater reliability based on SSI-4 was sufficiently high (93.8% agreement, Kappa = .89).

### Stimuli development

The stimuli used in the present study were identical to those described in Coalson and Byrd [41] and are provided in Table 2 for reference. Stimuli were comprised of 12 bisyllabic nonwords with CVCCVC word shape (C: consonant, V: vowel). As described in our prior study, each bisyllabic nonword was constructed to control for multiple linguistic, phonetic, and phonological factors known or thought to influence the speed, accuracy, and/or fluency of verbal response in AWS and AWNS. These factors include word-likeness [55], segment and biphone phonotactic probability [56], neighborhood density and frequency (e.g., [56], [57]), phonetic complexity (e.g., [58], cf. [59]), frequency of each syllable (e.g., [60]), orthographic transparency (e.g., [61]), uniqueness point (e.g., [62]), and syllable boundary clarity (see [63]).

**Table 2:** Target stimuli with associated foils across 12 experimental blocks.

Block	Trochaic	Iambic	Foil 1	Foil 2	Foil 3
1	/'fӕz.mul/	/fӕz.'mul/	/vim/	/zof/	/ʃəl/
2	/'zӕl.ʃov/	/zӕl.'ʃov/	/vif/	/miʃ/	/ləz/
3	/'ʃiv.lom/	/ʃiv.'lom/	/vuz/	/fəʃ/	/mɛl/
4	/'viʃ.fuz/	/viʃ.'fuz/	/ʃɛv/	/zom/	/laf/
5	/'lam.vef/	/lam.'vef/	/fɛʃ/	/miv/	/zol/
6	/'muf.zoʃ/	/muf.'zoʃ/	/faz/	/vim/	/ʃəl/
7	/'foʃ.vul/	/foʃ.'vul/	/ʃaz/	/zɪf/	/miv/
8	/'lev.mof/	/lev.'mof/	/vəl/	/faʃ/	/zim/
9	/'mӕz.fuv/	/mӕz.'fuv/	/vɛf/	/ʃom/	/zel/
10	/'ʃɛm.liz/	/ʃɛm.'liz/	/fuʃ/	/zev/	/mӕl/
11	/'vul.ziʃ/	/vul.'ziʃ/	/ʃaf/	/fɛv/	/lom/
12	/'zɪf.ʃom/	/zɪf.'ʃom/	/vul/	/feʃ/	/məz/

Values determined using databases and/or criteria detailed in Coalson and Byrd [41].

### Talker-Stress Groups

Participants in each talker group (AWS = 26; AWNS = 26) were randomly assigned to a stress condition, wherein half of each group heard all 12 nonword stimuli with trochaic stress, and the remaining half heard the same nonword stimuli with iambic stress. Each participant was exposed to nonword stimuli with only one stress pattern (trochaic or iambic), rather than each participant being exposed to both, to eliminate the potential for reduced accuracy during second exposure due to re-learning the same phonemic sequence with a different stress pattern. For example, phonemic and stress-assignment accuracy when producing iambic targets (e.g., *fazMOOL*) may have been lower if the same phonemic sequence was previously learned and produced with language-dominant trochaic stress patterns (e.g., *FAZmool*). Thus, in total, there were four distinct Talker-Stress Groups (AWNS-Trochaic, AWNS-Iambic, AWS-Trochaic, AWS-Iambic; *n* = 13 per group; eight males and five females per group; age range: 18 to 36 years across groups [*p* = .41]). Inclusionary and exclusionary criteria for participants in each Talker-Stress Group are provided in S1 Appendix.

### Procedure

**Fig 2:**
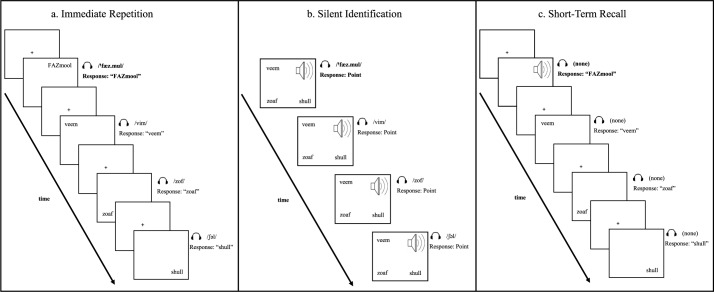
Schematic of the three-phase training task within one experimental block (e.g., /'fӕz.mul/). Bisyllabic nonword target depicted in bold font. *Immediate Repetition* (a) and *Short-Term Recall* (c) phases correspond with the immediate repetition and short-term recall tasks described in the present study. Auditory and orthographic cues for nonword target were present during completion of the *Immediate Repetition* (a) phase. Orthographic cues, but not auditory cues, were removed for nonword target during completion of the *Silent Identification* (b) phase. Both auditory and orthographic cues for nonword target were removed during completion of the *Short-Term Recall* (c) phase.

The experimental paradigm used in this study was designed to examine the accuracy of AWNS and AWS when producing trochaic and iambic nonwords from memory in tasks when they were and were not presented with auditory-orthographic cues. The three-phase procedure was derived from the training paradigm originally developed by Levelt and colleagues ([60], [64], [65]). Our modified version of this three-phase task is detailed in Fig 2 and was used by Coalson and Byrd [41] to train AWNS and AWS to generate a target nonword upon presentation of a 2 in x 2 in speaker icon positioned in one of four corners of a computer screen. The structure of this paradigm allowed assessment of (a) immediate repetition of nonwords in the presence of auditory-orthographic cues during the first phase (Fig 2A), and (b) recall of these nonwords in the absence of auditory-orthographic cues during the third phase (Fig 2C). See https://digitalcommons.lsu.edu/comd_pubs/1 for a short video demonstration of this unique three-phase training task.

The following will describe the sequence of events within each phase (i.e., *immediate repetition*, *silent identification*, and *short-term recall*) of a single experimental block (see Fig 2A, 2B and 2C). A single experimental block (e.g., Block 1) contained one target nonword (e.g., /fӕz.mul/) and the three monosyllabic foils (e.g., [vim, zof, ʃəl]) listed in Table 2. Monosyllabic foils were necessary to minimize anticipation strategies by the participant and prevent the advantage of motoric priming due to consecutive productions. Single-syllable foils also prevented potential priming of a specific stress pattern prior to producing the bisyllabic target nonword. All participants completed all 12 blocks, one per target nonword, and each block consisted of the three-phase sequence depicted in Fig 2, further detailed in S2 Appendix, and demonstrated in the supplemental video available at https://digitalcommons.lsu.edu/comd_pubs/1.

#### Immediate repetition

During the immediate repetition phase, participants were instructed to repeat each nonword target and nonword foil immediately after simultaneous auditory and orthographic cues (see Fig 2A). Target nonwords (e.g., /fӕz.mul/) were presented in one designated corner (e.g., top right), and the three monosyllabic foils (e.g., [vim, zof, ʃəl]) were presented in the remaining three corners. Audio samples of each nonword were presented via headphones as each nonword appeared, one-by-one, in randomized order. Participants assigned to the trochaic condition (i.e., AWNS-Trochaic, AWS-Trochaic) heard the target nonword with trochaic stress (e.g., /'fӕz.mul/) and viewed an orthographic representation with capitalized letters to indicate stress on the first syllable (e.g., *FAZmool*). Participants assigned to the iambic condition (i.e., AWNS-Iambic, AWS-Iambic) heard the target nonword with iambic stress (e.g., /fӕz.'mul/) and viewed an orthographic representation with capitalized letters to indicate stress on the second syllable (e.g., *fazMOOL*). Each nonword was preceded by an orienting cross in the center of the screen for 500 ms, and advancement to the following slide was self-paced to accommodate for disfluent speech. Verbal responses were scored offline for fluency and accuracy.

The target nonword (e.g., /fӕz.mul/) and each monosyllabic foil (e.g., [vim, zof, ʃəl]) were each presented four times during the immediate repetition phase, resulting in 16 total immediate repetition trials within a single experimental block. That is, each participant was provided four attempts to repeat the target nonword within a single block (in addition to four repetitions of each monosyllabic foil). Upon completion of the immediate repetition phase (Fig 2A), the participant began the silent identification phase (Fig 2B).

#### Silent identification

During the silent identification phase, participants were instructed to point to the corner of the computer screen that corresponded with each nonword (e.g., [fӕz.mul, vim, zof, ʃəl]) presented via headphones. However, the orthographic cue for the target nonword (e.g., *FAZmool*) was replaced by a 2 in x 2 in speaker icon (see Fig 2B) in the designated corner used during the immediate repetition phase (e.g., top right). All monosyllabic foils remained visible in written form in their designated corners. The speaker icon and three orthographic foils were displayed in each corner of the screen continuously throughout the silent identification phase. Audio samples for all stimuli (one target nonword, three nonword foils) were presented, one-by-one, via headphones four times in randomized order at 1-second intervals, resulting in 16 trials per block. The silent identification phase required no verbal response and lasted 16 seconds (i.e., 1 second per trial) before the participant advanced to the short-term recall phase (Fig 2C).

#### Short-term recall

During the short-term recall phase, participants were instructed to say the nonword aloud when either the speaker icon or written foil appeared in its designated corner. No auditory or orthographic input was provided for the target nonword. That is, participants produced the target nonword (e.g., /fӕz.mul/) upon presentation of the speaker icon in its designated corner (e.g., top right), and in the absence of auditory and orthographic cues. The speaker icon and three orthographic foils were presented, one-by-one, in randomized order in the same corners established during the immediate repetition and silent identification phases (see Fig 2C). Similar to immediate repetition, each trial was preceded by an orienting cross in the center of the screen for 500 ms, and advancement to the next slide was self-paced to accommodate for disfluent speech. Verbal responses were scored offline for fluency and accuracy.

The target nonword (e.g., /fӕz.mul/, represented by the speaker icon) and each monosyllabic foil (e.g., [vim, zof, ʃəl], represented in written form) were presented four times in randomized order during the short-term recall phase of an experimental block, resulting in 16 short-term recall trials within a single experimental block. That is, each participant was provided four attempts to produce the target nonword (e.g., /fӕz.mul/) within a single block (in addition to four productions of each monosyllabic foil). Upon completion of the short-term recall phase (Fig 2C), the participant began a new experimental block (e.g., Block 2) with a different target nonword (e.g., /zӕl.ʃov/) and different foils (e.g., [vif, miʃ, ləz]).

Each participant completed all 12 experimental blocks during the second 90-minute session. This resulted in 192 immediate repetition trials and 192 short-term recall trials per participant (i.e., 16 trials per block x 12 blocks). Of these trials, each participant produced a total of 48 target nonwords during the immediate repetition phase (i.e., 4 presentations of a single target nonword within each block x 12 blocks) and 48 target nonwords during the short-term recall phase (i.e., four presentations of a single target nonword within each block x 12 blocks). Trials produced by each participant in response to monosyllabic foils during immediate repetition (i.e., 12 within each block x 12 blocks) and short-term recall (i.e., 12 within each block x 12 blocks) were not scored or included during analysis. Therefore, each Talker-Stress Group provided 624 tokens during immediate repetition (48 productions x 13 participants per group) and 624 tokens during short-term recall (48 productions x 13 participants per group).

#### Data scoring

Two trained undergraduate students coded the accuracy of verbal responses. A phonemic error was defined as production of a target nonword with one or more phonemic errors during production (i.e., substitution, deletion, and/or insertion). Inter-rater reliability during immediate repetition and short-term recall was established based on approximately one-third of the 52 usable participants (*n* = 14; AWNS = 7, AWS = 7). Production errors for participant responses were scored with a high level of reliability (i.e., 97.5% agreement; Kappa = .871, *p* < .001).

#### Excluded tokens

Data were removed from final analyses if the following criteria were met:

- No Response: participant provided no verbal response or initiated verbal response more than 3000 ms after presentation of cue.
- Stuttered response: verbal response contained a stuttering-like disfluency (i.e., sound-syllable repetition, audible sound prolongation, inaudible sound prolongation, or any combination of these disfluencies).
- Disfluent response: verbal response contained a typical disfluency (i.e., revision, interjection, or any combination of these disfluencies).
- Stress error: verbal response with stress that deviated from the target metrical pattern (i.e., iambic production for trochaic target, trochaic production for iambic target, or production of neutral stress pattern for either target stress pattern).
- Error combination: combination of more than one of the following types of responses–phonemic error, stress error, stuttered response, disfluent response, and/or no response.
- Technical error: verbal response could not be coded due to audio-video difficulties (e.g., inaudible response, audio-video interruption) or non-speech events (e.g., yawn, cough).

S3 Appendix provides a detailed summary of data exclusion for immediate repetition and short-term recall tasks. In sum, a total of 2,496 tokens were collected from all participants (i.e., AWNS-Trochaic: *n* = 624, AWNS-Iambic: *n* = 624, AWS-Trochaic: *n* = 624, AWNS-Iambic: *n* = 624) for each task. Based on these criteria, 127 (5.09%) tokens were excluded from the immediate repetition task, and 145 (5.81%) tokens were excluded from the short-term recall task. This resulted in 2,369 usable tokens for immediate repetition (AWNS-Trochaic: *n* = 606, AWNS-Iambic: *n* = 611, AWS-Trochaic: *n* = 566, AWNS-Iambic: *n* = 586) and 2,351 usable tokens for short-term recall (AWNS-Trochaic: *n* = 604, AWNS-Iambic: *n* = 609, AWS-Trochaic: *n* = 562, AWNS-Iambic: *n* = 576).

#### Analysis

To review, the purpose of the present study was to examine the verbal accuracy of AWS and AWNS during immediate repetition and short-term recall of nonwords that carried either trochaic or iambic stress. Half of each talker group (AWNS, *n* = 26; AWS, *n* = 26) were presented nonwords with trochaic stress (AWNS, *n* = 13; AWNS, *n* = 13), and the remaining half were presented the same nonword stimuli with iambic stress (AWNS, *n* = 13; AWS *n* = 13), resulting in four distinct Talker-Stress Groups (AWNS-Trochaic, AWS-Trochaic, AWNS-Iambic, AWS-Iambic).

To examine phonemic accuracy during immediate repetition and short-term recall, a multilevel mixed-model analysis was conducted using the procedure for SPSS (v. 24) described by Field [66]. Mean phonemic accuracy of the 12 nonwords served as the dependent variable (12 nonwords = 12 targets per participant). Talker Group (AWNS, AWS), Stress (Trochaic, Iambic), and Task (Immediate Repetition, Short-Term Recall) served as the fixed effects, as well as all two-way and three-way interactions between these terms. As noted, each participant provided multiple responses for a single target nonword within each task (i.e., four attempts per target during immediate repetition [Attempt 1, Attempt 2, Attempt 3, Attempt 4], four attempts per target during short-term recall [Attempt 1, Attempt 2, Attempt 3, Attempt 4]). Therefore, Attempt within Task was also included as repeated measures. Attempt within Task was not a variable of interest and, for this reason, no specific hypothesis was provided. See S4 Appendix for further detail regarding the statistical and theoretical rationale for inclusion of Attempt within Task as a random effect.

Satterthwaite approximation of standard error was applied to account for small sample size. All pairwise comparisons were conducted using sequential Bonferroni adjusted *p*-values. As recommended by Raudenbush and Liu [67], effect sizes (*d*) were estimated by *b* / (τ)^1/2^, where *b* is the regression coefficient and τ is the error variance of the random effect. To determine model fitness, AIC values were compared upon inclusion of each fixed effect (Talker Group, Stress, Task), followed by each two-way interaction term (Talker Group x Task, Talker Group x Stress, Stress x Task), the three-way interaction term (Talker Group x Stress x Task), and finally the repeated measures component (Attempt within Task). AIC values were compared upon inclusion of each to determine overall fitness of model. Reduced AIC values in each successive model indicated that model fitness was highest upon inclusion of all terms. Intraclass correlation coefficients (ICC) were calculated to assess proportion of variance in the response accuracy explained by within-participant and between-participant variance. See Table 3 for parameter estimates, AIC values, and ICC values across each model.

**Table 3:** Parameter estimates for the nine models examining the relationship between Talker Group, Task, and Stress upon phonemic accuracy of nonword production.

	M1	M2	M3	M4	M5	M6	M7	M8	M9^†^
*Fixed Components*									
Intercept	11.15***	10.81***	10.58***	9.83***	9.53***	9.41***	9.15***	9.04***	9.04***
Talker Group		.67**	.67**	.67**	1.27***	1.51***	1.51***	1.73***	1.74***
Stress			.48*	.48*	.48*	.72*	1.25***	1.46***	1.46***
Task				1.49***	2.09***	2.09***	2.61***	2.83***	2.84***
Talker Group x Task					-1.20***	-1.20***	-1.20***	-1.64***	-1.64***
Talker Group x Stress						-.49	-.49	-.92*	-.93*
Stress x Task							-1.05***	-1.48***	-1.48***
Talker Group x Stress x Task								.87**	-.93**
*Random components*									
Intercept	.56***	.46***	.41***	.49***	.50***	.50***	.51**	.51***	.05***
Residual	1.48***	1.48***	1.48***	.86***	.75***	.75***	.68***	.67***	
Residual (IR, Attempt 1)									.09***
Residual (IR, Attempt 2)									.07***
Residual (IR, Attempt 3)									.07***
Residual (IR, Attempt 4)									.05**
Residual (STR, Attempt 1)									2.01***
Residual (STR, Attempt 2)									1.94***
Residual (STR, Attempt 3)									2.01***
Residual (STR, Attempt 4)									1.91**
ICC	.27	.24	.22	.36	.40	.41	.43	.43	IR [.36 to .50] STR [.02 to .03]
AIC	1422.34	1415.06	1411.52	1212.97	1167.85	1166.39	1128.17	1121.35	866.62

*Note*. IR: Immediate Repetition, STR: Short-Term Recall.

* *p* < .05

** *p* < .01

*** *p* < .001

^†^ Fitted model selected for analysis of phonemic accuracy.

## Results

### Phonemic accuracy

Multilevel linear mixed model analysis revealed a significant main effect of Talker Group *F*(1, 185) = 32.44, *p* < .001, *d* = .34, with fewer correct nonwords produced by AWS (*M* = 10.82, *SE* = 0.08) than AWNS (*M* = 11.48, *SE* = 0.08). A significant main effect was also detected for Stress *F*(1, 185) = 16.60, *p* < .001, *d* = .29, with iambic nonwords produced less accurately (*M* = 10.91, *SE* = 0.08) than trochaic nonwords (*M* = 11.39, *SE* = 0.08). A significant main effect was also detected for Task *F*(1, 206) = 228.48, *p* < .001, *d* = .55, with fewer accurate nonwords produced during short-term recall (*M* = 10.40, *SE* = 0.10) than immediate repetition (*M* = 11.90, *SE* = 0.04).

Three significant two-way interactions were also revealed during analysis. Overall, AWS produced fewer accurate nonwords during short-term recall (*M* = 9.77, *SE* = 0.14) than AWNS (*M* = 11.04, *SE* = 0.14; Talker Group x Task: *F*(1, 206) = 37.55, *p* < .001, *d* = .32). AWS also produced fewer accurate iambic nonwords (*M* = 10.45, *SE* = 0.12) than trochaic nonwords (*M* = 11.18, *SE* = 0.12; Talker Group x Stress: *F*(1, 185) = 4.42, *p* = .037, *d* = .18). Overall, all participants produced fewer accurate iambic nonwords during short-term recall (*M* = 9.90, *SE* = .14) than immediate repetition (*M* = 11.92, *SE* = 0.05; Stress x Task: *F*(1, 206) = 27.79, *p* < .001, *d* = .29).

**Fig 3:**
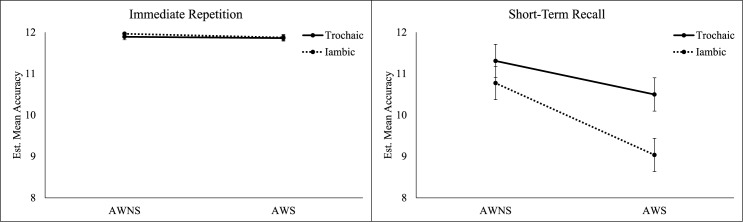
Trochaic and iambic nonwords produced without phonemic error by adults who stutter (AWS) and adults who do not stutter (AWNS) during immediate repetition and short-term recall tasks.

However, a significant three-way interaction was detected between Talker Group, Stress, and Task *F*(1, 206) = 4.80, *p* = .030, *d* = .17. As depicted in Fig 3, AWS recalled both trochaic nonwords (*M* = 10.50, *SE* = .20) and iambic nonwords (*M* = 9.04, *SE* = .20) with significantly lower phonemic accuracy during short-term recall than AWNS (trochaic: *M* = 11.31, *SE* = .20, *p* = .005; iambic: *M* = 10.77, *SE* = .20, *p* < .001). During short-term recall, AWS produced iambic nonwords with significantly lower accuracy than trochaic nonwords (*p* < .001). No significant difference in accuracy between trochaic and iambic nonwords was found for AWNS during short-term recall (*p* = .065). A summary of model estimates for all main effects and interaction terms is provided in Table 4.

**Table 4:** Regression coefficients (*b*), standard error of the coefficient (*SE*_*b*_), confidence intervals (*CI*), and effect sizes (*d*) of multilevel models estimates of phonemic accuracy of adults who do and do not stutter (Talker Group) when producing trochaic and iambic nonwords (Stress) during immediate repetition and short-term recall (Task).

	*b*	*SE*_*b*_	95% *CI*	*d*
Talker Group	1.73***	0.32	1.09 to 2.38	.34
Stress	1.46***	0.32	0.82 to 2.11	.29
Task	2.84***	0.16	2.51 to 3.14	.55
Talker Group x Task	-1.64***	0.23	-2.08 to -1.19	.32
Talker Group x Stress	-0.93*	0.46	-1.84 to -0.01	.18
Stress x Task	-1.48***	0.23	-1.93 to -1.04	.29
Talker Group x Stress x Task	0.87**	0.32	0.24 to 1.50	.17

Note.

*p < .05

**p < .01

***p < .001.

Effect size *d* = *b* / (τ)^1/2^, where τ is the error variance of the random effect.

### Stress accuracy

Stress-assignment errors were not the primary focus of the present study and were relatively infrequent across tasks (i.e., 44 total during immediate repetition, 40 total during short term recall; 1.75% of all usable data; see S3 Appendix). In addition, no meaningful statistical analysis could be conducted, as model fitness was not improved beyond between- and within-participant variability. That is, AIC values remained lowest for the intercept-only model (396.94) compared to models which included any combination of fixed factors (range: 397.15 to 400.81). Nonetheless, descriptive data of stress accuracy by each group may be informative based on the number of increased stress-assignment errors produced by individuals who stutter in previous studies ([40], [41]). Overall, AWNS produced fewer stress-assignment errors during immediate repetition (Trochaic: 6 of 606 [0.99%]; Iambic: 2 of 609 [0.33%]) and short-term recall (Trochaic: 6 of 604 [0.99%]; Iambic: 0 of 609 [0.00%]) than AWS during immediate repetition (Trochaic: 8 of 566 [1.41%]; Iambic: 28 of 586 [4.78%]) and short-term recall (Trochaic: 17 of 562 [3.02%]; Iambic: 17 of 576 [2.95%]).

## Discussion

Previous investigations of phonological working memory in AWS and AWNS required participants produce nonwords with simple, trochaic stress immediately after a presentation of either an auditory or an orthographic cue. The present study examined whether phonological working memory abilities of AWS differ from AWNS under more challenging conditions. Two methodological factors were manipulated (i.e., stimuli stress, cueing methods) which may have minimized phonological demand in previous studies and, as a result, underestimated the vulnerability of phonological working memory in AWS. Participants in the present study were presented with 12 bisyllabic nonwords that differed only by stress assignment (i.e., high-frequency trochaic stress, low-frequency iambic stress). Participants produced these nonwords during two tasks: 1) an immediate repetition task, in which the speaker produced the target after an auditory-orthographic cue, and 2) a short-term recall task, in which participants produced the target in the absence of auditory-orthographic input.

A significant main effect was found for Talker Group, Stress, and Task. Overall, AWS were less accurate than AWNS, and all participants were less accurate when producing iambic nonwords or producing nonwords in the absence of auditory-orthographic cues. All two-way interactions were significant, indicating overall poorer accuracy for AWS than AWNS when producing iambic nonwords, or when auditory-orthographic cues had been removed. However, a significant three-way interaction demonstrated that decreased accuracy by AWS was limited to the most challenging condition. That is, AWS were less accurate than AWNS only when producing iambic nonwords *and* when auditory-orthographic cues were removed. Findings suggest that phonological working memory in AWS may be more susceptible to phonological demand–even when producing short, bisyllabic targets. These data introduce the possibility that previous studies may have underestimated phonological working memory weaknesses in AWS due to predominately trochaic stimuli and production of these stimuli immediately after an auditory or orthographic cue.

### Subvocal rehearsal

One critical consideration when interpreting reduced accuracy of iambic nonwords during short-term recall is the potential limitations of subvocal rehearsal in AWS. Although weaknesses in subvocal rehearsal in AWS are consistent with previous research ([23], [24]), we cannot say with certainty that subvocal rehearsal was actively employed in the present study by all participants for all trials. Moreover, the nature of the short-term recall task in the present study differed from recall paradigms used in previous studies (e.g., [21], [43]). For example, participants were provided four additional opportunities to verify the accuracy of the target nonword prior to recall during the intervening silent identification phase [Fig 2B] (however, see [26] for discussion of the disproportionate benefit of multiple nonword exposures to AWNS versus AWS). Nevertheless, assuming that subvocal rehearsal was employed to some extent during short-term recall, it was still less beneficial for AWS, particularly for iambic nonwords.

Baddeley’s [25] description of phonological working memory provides at least two possible predictions for why iambic stress patterns may impede subvocal rehearsal in AWS more than trochaic stress patterns. From a motoric perspective, it is possible the motor templates thought to support subvocal rehearsal may have been less stable for iambic nonwords for AWS. In AWNS, iambic patterns undergo considerable motoric refinement with age and, as a result, are produced with *greater* motoric coordination than trochaic patterns (e.g., [68], [69]). If AWS exhibit a developmental ‘lag’ in speech motor coordination across the lifespan (e.g., [4], [70]), iambic motor templates may remain less stable in AWS than AWNS and provide weaker re-activation of phonological sequences during rehearsal. From a linguistic perspective, AWS may exhibit even greater difficulty accessing infrequent metrical patterns than AWNS (e.g., [36]) as observed in AWS for infrequent segmental patterns [21]. If less frequent metrical patterns are stored within the lexical representation, as proposed by Levelt and colleagues [71], simultaneous retrieval of segmental information *and* infrequent metrical properties in AWS may have slowed re-activation of iambic targets as opposed to targets with a default, trochaic structure (see Fig 1A). Additional modifications to the short-term recall task used in this study are warranted to discern the independent contribution of motoric and linguistic processing during subvocal rehearsal of iambic nonwords in AWS (e.g., iambic recall accuracy in the presence of articulatory suppression [20]). Nonetheless, the present study provides preliminary data that less-frequent metrical stress may impair subvocal rehearsal in AWS, and these differences may be attributable to either the recruitment of less stable motoric templates, weaker re-encoding after recruitment of motor templates, or perhaps both.

### Short-term recall and strategy use

Due to the increased demand during short-term recall, individual differences in strategy use may have influenced recall accuracy. Strategy mediation [72] data suggest that application of various recall strategies during short-term memory tasks other than subvocal rehearsal–such as interactive imagery or lexical association–may predict performance more so than underlying working memory abilities. Strategy use was not the primary focus of the study, and participants were not given explicit instructions to employ or refrain from memory strategies. However, post-session feedback collected from participants indicated that very few conscious strategies were actively employed by AWS or AWNS to improve recall. Of the 52 participants, only seven (13.46%) reported active strategy use to recall target nonwords (AWNS-Trochaic, *n* = 2; AWS-Trochaic, *n* = 1, AWNS-Iambic, *n* = 1; AWS-Iambic, *n* = 3). Of these seven, subvocal rehearsal was reported most often (*n* = 4), followed by lexical association (*n* = 2) and visualization of written form (*n* = 1). These data should be interpreted with caution as data were collected after completion of all 12 experimental blocks. As noted by Dunlosky and Kane [73], retroactive free-recall of strategy use during working memory tasks is prone to several caveats. For example, participants may (a) develop inaccurate reports that do not reflect actual strategies used during individual trials, (b) simply not remember how accurate recall was achieved on individual trials, or (c) have no knowledge of specific “strategies” and fail to report any if asked. Our data are consistent with these caveats, as a majority of participants (86.54%) reported no conscious use of any strategy. Based on these limited post-hoc data, we cannot determine with any certainty whether strategy use differed between groups or disproportionately benefitted AWS or AWNS. Future investigations should require participants to report strategies used to improve accuracy, preferably set-by-set, and examine the benefit of these strategies to AWS relative to AWNS.

### Clinical implications

In terms of clinical implications, the present data suggest that modifying stimuli stress and cueing methods of existing nonword repetition tasks may improve their diagnostic and prognostic value when assessing individuals who stutter. Two of the most frequently used nonword repetition tasks in the stuttering literature (*Nonword Repetition Task* [74], *Children’s Test of Nonword Repetition* [75]) require participants to repeat nonword targets immediately after auditory cues, and include primarily trochaic nonword targets (e.g., 8 of 12 items, 31 of 40 items, respectively). In response to Smith and Weber’s [76] call for improved measures to assess the likelihood of persistence in children who stutter, we propose that the modifications to the standard nonword repetition task used in the present study be considered. That is, manipulation of stimuli stress and cueing methods may yield even greater differences when applied to children who stutter, who exhibit less accurate repetition of 2-syllable nonwords even *with* the benefit of auditory cues and predominately trochaic stimuli ([16], [17]). For example, Spencer and Weber-Fox [8] found that children who persist in stuttering repeat nonwords less accurately compared to children who recover from stuttering as syllable length increased. The authors also note, however, a large number of children in the persistent cohort repeated 3-syllable (17 of 19; 89%) and 4-syllable (12 of 19; 63%) targets with accuracy similar to the recovered cohort. Based on our findings, systematic increase of metrical complexity of nonword targets, as well as segmental length, may better discriminate children who persist from those who recover. Inclusion of a brief delay before verbal response may further separate groups by allowing the benefit of auditory-orthographic priming to subside prior to response. These claims remain speculative, and additional research in younger children who stutter is warranted to verify whether the diagnostic and prognostic utility of nonword repetition is improved with these two relatively simple modifications.

## Conclusion

The purpose of the present study was to examine the contribution of metrical stress and nonword cueing effects on phonological working memory abilities in AWS. Our findings indicate that less-frequent metrical properties and removal of phonological cues before response significantly impede retention of segmental sequences in AWS. These results suggest greater weaknesses in phonological processing in AWS than previously reported, and underscore the importance of carefully considering task-related demands in future studies of phonological working memory.

## Supporting information

S1 Appendix Number of recruited participants per Talker-Stress Group who did not meet inclusionary and exclusionary criteria. AWNS: adults who do not stutter; AWS: adults who stutter. Speech diagnoses included diagnosed or observed articulatory and phonological disturbances other than stuttering. ^1^English proficiency based on 7-point self-rating scale in Language History Questionnaire ([49], [50]). ^2^ Binaural pure tone hearing screening [47]. ^3^Visual acuity screening [48].
(DOCX)

S2 Appendix Structure of a training task within a single block. Three-phase training procedures detailed in Coalson and Byrd [41] and based on training paradigms described by Levelt and colleagues ([60], [64], [65]).
(DOCX)

S3 Appendix Number of responses excluded per Talker-Stress Group during immediate repetition and recall tasks. AWNS: adults who do not stutter; AWS: adults who stutter. Percentage based on initial data corpus for each task (immediate repetition, short-term recall).
(DOCX)

S4 Appendix Theoretical and statistical rationale for attempt within Task as random effect. (DOCX)

S1 Data Phonemic accuracy data. (XLSX)
