# Supplementary material for: Nonword repetition in adults who stutter: The effects of stimuli stress and auditory-orthographic cues
Source: PLoS One. 2017 Nov 29;12(11):e0188111. doi: 10.1371/journal.pone.0188111 (PMC5706734; doi:10.1371/journal.pone.0188111)
Supplement: S1 Appendix — AWNS: adults who do not stutter; AWS: adults who stutter. Speech diagnoses included diagnosed or observed articulatory and phonological disturbances other than stuttering. 1English proficiency based on 7-point self-rating scale in Language History Questionnaire ([49], [50]). 2 Binaural pure tone hearing screening [47]. 3Visual acuity screening [48]. (DOCX) [file pone.0188111.s001.docx]

|  |  | Inclusionary | | | |  | Exclusionary | |  |
| --- | --- | --- | --- | --- | --- | --- | --- | --- | --- |
|  | Number Recruited | Age (18+) | No current medical or speech concerns | No current use of antipsychotic medication | Native English proficiency^1^ |  | Failed hearing^2^ or vision screenings^3^ | Removed to balance age and gender of groups | Final cohort |
| *Trochaic Condition* | | | | | | | | | |
| AWNS | 23 | 0 | 1 | 0 | 2 |  | 1 | 6 | 13 |
| AWS | 22 | 0 | 0 | 1 | 5 |  | 0 | 3 | 13 |
| Total | 45 | 0 | 1 | 1 | 7 |  | 1 | 9 | 26 |
|  |  |  |  |  |  |  |  |  |  |
| *Iambic Condition* | | | | | | | | | |
| AWNS | 20 | 0 | 0 | 1 | 2 |  | 0 | 4 | 13 |
| AWS | 22 | 0 | 0 | 1 | 4 |  | 0 | 4 | 13 |
| Total | 42 | 0 | 0 | 4 | 6 |  | 0 | 8 | 26 |
