# Supplementary material for: Nonword repetition in adults who stutter: The effects of stimuli stress and auditory-orthographic cues
Source: PLoS One. 2017 Nov 29;12(11):e0188111. doi: 10.1371/journal.pone.0188111 (PMC5706734; doi:10.1371/journal.pone.0188111)
Supplement: S2 Appendix — Three-phase training procedures detailed in Coalson and Byrd [41] and based on training paradigms described by Levelt and colleagues ([60], [64], [65]). (DOCX) [file pone.0188111.s002.docx]

| Nonword Stimuli | Auditory  Cue | Auditory  Presentation | Visual  Cue | Visual  Presentation | Participant Response | Order | No. of Exposures |
| --- | --- | --- | --- | --- | --- | --- | --- |
| 1. *Immediate Repetition* | | | | | | | |
| Target | Heard target | Simultaneous with Visual Cue | Orthographic, Corner A | Corner A | Repeat aloud | Random | 4 |
| Foil 1 | Heard foil | Simultaneous with Visual Cue | Orthographic, Corner B | Corner B | Repeat aloud | Random | 4 |
| Foil 2 | Heard foil | Simultaneous with Visual Cue | Orthographic, Corner C | Corner C | Repeat aloud | Random | 4 |
| Foil 3 | Heard foil | Simultaneous with Visual Cue | Orthographic, Corner D | Corner D | Repeat aloud | Random | 4 |
| 1. *Silent Identification* | | | | | | | |
| Target | Heard target | Simultaneous with Visual Cue | Neutral Icon, Corner A | Corners A-D | Point to corner | Random | 4 |
| Foil 1 | Heard foil | Simultaneous with Visual Cue | Orthographic, Corner B | Corners A-D | Point to corner | Random | 4 |
| Foil 2 | Heard foil | Simultaneous with Visual Cue | Orthographic, Corner C | Corners A-D | Point to corner | Random | 4 |
| Foil 3 | Heard foil | Simultaneous with Visual Cue | Orthographic, Corner D | Corners A-D | Point to corner | Random | 4 |
| 1. *Short-Term Recall* | | | | | | | |
| Target | None | None | Neutral Icon, Corner A | Corner A | Say nonword | Random | 4 |
| Foil 1 | None | None | Orthographic, Corner B | Corner B | Say nonword | Random | 4 |
| Foil 2 | None | None | Orthographic, Corner C | Corner C | Say nonword | Random | 4 |
| Foil 3 | None | None | Orthographic, Corner D | Corner D | Say nonword | Random | 4 |
