# Supplementary material for: Nonword repetition in adults who stutter: The effects of stimuli stress and auditory-orthographic cues
Source: PLoS One. 2017 Nov 29;12(11):e0188111. doi: 10.1371/journal.pone.0188111 (PMC5706734; doi:10.1371/journal.pone.0188111)
Supplement: S3 Appendix — AWNS: adults who do not stutter; AWS: adults who stutter. Percentage based on initial data corpus for each task (immediate repetition, short-term recall). (DOCX) [file pone.0188111.s003.docx]

|  |  |  |  |  |  |  |  |  |  |
| --- | --- | --- | --- | --- | --- | --- | --- | --- | --- |
| *Immediate Repetition* | AWNS-Trochaic |  | AWNS-Iambic |  | AWS-Trochaic |  | AWS-Iambic |  | Total |
| ***N*** | **624** |  | **624** |  | **624** |  | **624** |  | **2496** |
| No response | 1 [0.04%] |  | 0 [0.04%] |  | 2 [0.08%] |  | 0 [0.00%] |  | 3 [0.12%] |
| Stuttered response | 0 [0.00%] |  | 1 [0.04%] |  | 26 [1.04%] |  | 7 [0.28%] |  | 34 [1.36%] |
| Disfluent response | 6 [0.24%] |  | 3 [0.12%] |  | 5 [0.20%] |  | 1 [0.04%] |  | 15 [0.60%] |
| Stress error | 6 [0.24%] |  | 2 [0.08%] |  | 8 [0.32%] |  | 28 [1.12%] |  | 44 [1.76%] |
| Error combo | 0 [0.00%] |  | 0 [0.00%] |  | 6 [0.24%] |  | 0 [0.00%] |  | 6 [0.24%] |
| Technical error | 5 [0.20%] |  | 7 [0.28%] |  | 11 [0.40%] |  | 2 [0.08%] |  | 25 [1.00%] |
| Total Excluded | 18 [0.72%] |  | 13 [0.52%] |  | 58 [2.33%] |  | 38 [1.52%] |  | 127 [5.09%] |
| **Usable** | **606 [24.28%]** |  | **611 [24.20%]** |  | **566 [22.68%]** |  | **586 [23.48%]** |  | **2369 [94.91%]** |
|  |  |  |  |  |  |  |  |  |  |
| **Accurate tokens** | **600 [24.04%]** |  | **609 [24.40%]** |  | **558 [22.36%]** |  | **579 [23.20%]** |  | **2346 [93.99%]** |
| **Tokens with phonemic error** | **6 [0.24%]** |  | **2 [0.08%]** |  | **8 [0.32%]** |  | **7 [0.28%]** |  | **23 [0.92%]** |
|  |  |  |  |  |  |  |  |  |  |
| *Short-Term Recall* | AWNS-Trochaic |  | AWNS-Iambic |  | AWS-Trochaic |  | AWS-Iambic |  | Total |
| ***N*** | **624** |  | **624** |  | **624** |  | **624** |  | **2496** |
| No response | 1 [0.04%] |  | 2 [0.08%] |  | 5 [0.20%] |  | 3 [0.12%] |  | 11[0.44%] |
| Stuttered response | 0 [0.00%] |  | 1 [0.04%] |  | 22 [0.88%] |  | 21 [0.84%] |  | 44 [1.76%] |
| Disfluent response | 5 [0.20%] |  | 2 [0.08%] |  | 5 [0.20%] |  | 3 [0.12%] |  | 15 [0.60%] |
| Stress error | 6 [0.64%] |  | 0 [0.00%] |  | 17 [0.68%] |  | 17 [0.68%] |  | 40 [1.60%] |
| Error combo | 1 [0.04%] |  | 5 [0.20%] |  | 5 [0.20%] |  | 4 [0.16%] |  | 15 [0.60%] |
| Technical error | 7 [0.28%] |  | 5 [0.20%] |  | 8 [0.32%] |  | 0 [0.00%] |  | 20 [0.80%] |
| Total Excluded | 20 [0.80%] |  | 15 [0.60%] |  | 62 [2.48%] |  | 48 [1.92%] |  | 145 [5.81%] |
| **Usable** | **604 [23.20%]** |  | **609 [24.40%]** |  | **562 [22.51%]** |  | **576 [23.08%]** |  | **2351 [94.19%]** |
|  |  |  |  |  |  |  |  |  |  |
| **Accurate tokens** | **586 [22.77%]** |  | **544 [21.80%]** |  | **484 [19.39%]** |  | **431 [17.27%]** |  | **2027 [81.21%]** |
| **Tokens with phonemic error** | **36 [1.44%]** |  | **65 [2.60%]** |  | **78 [3.13%]** |  | **145 [5.81%]** |  | **324 [12.98%]** |
