# Supplementary material for: Nonword repetition in adults who stutter: The effects of stimuli stress and auditory-orthographic cues
Source: PLoS One. 2017 Nov 29;12(11):e0188111. doi: 10.1371/journal.pone.0188111 (PMC5706734; doi:10.1371/journal.pone.0188111)
Supplement: S4 Appendix — (DOCX) [file pone.0188111.s004.docx]

The rationale for including Attempt within Task as a random component was twofold. From a statistical perspective, Attempt within Task was considered as a repeated measure because each participant provided 4 production attempts [i.e., Attempt] during 2 tasks [Task]. Verbal responses collected across multiple time points from the same participant introduced potential dependency in data. Thus, inclusion of Attempt within Task as random effects was necessary to account for the proportion of variance attributable to correlation of residuals.

From a theoretical perspective, it was reasonable to assume that the order in which verbal responses were provided within a block [Attempt 1, Attempt 2, Attempt 3, Attempt 4] may account for a proportion of accuracy between or within participants. It was also reasonable to assume that this order effect may differ based on the demands of the Task [immediate repetition – lower; short-term recall – higher]. Thus, inclusion of Attempt within Task as a random effect in the model allowed researchers to account for potential order effects between individual participants when measuring verbal accuracy of each group across tasks.

This latter point is supported by the model-fitting data and ICC values provided in Table 3 (p. 20). Based on the intercept-only model [M1], approximately 27% (ICC = .27) of production accuracy was explained by differences between participants alone. When compared to a model which only includes Attempt within Task as random effects, in addition to intercept (provided in the table below), increased ICC values indicate that a considerable proportion of accuracy was accounted for by order effects. However, the influence of order effect differed within each task. As indicated in the table below, the proportion of variance accounted for by order effects between participants was *higher* for immediate repetition [ICC values ranged from .31 to .44 across all attempts] but *lower* for short-term recall [ICC values decreased to .01 across all attempts]. This can be interpreted to suggest that order effects contributed more so to individual participants’ responses during immediate repetition (i.e., individual participants varied in their response to order effect), but minimally during short-term recall (i.e., participants responded with greater similarity across multiple attempts). One reason for this pattern may be that if a participant responded accurately (or inaccurately) upon *initial* attempt during the short-term recall, their accuracy did not change on subsequent attempts because no auditory or orthographic cues were provided to verify accuracy. This general pattern holds even after inclusion of the three fixed effects and their interaction terms in Model 9 [M9] reported in the manuscript (p. 20). As shown in Table 3, ICC values for immediate repetition range from .36 to .50 across attempts, while ICC values for short-term recall range from .02 to .03 across attempts.

|  | Intercept-only model [M1] | Model with intercept and Attempt with Task as random coefficients |
| --- | --- | --- |
| *Fixed Components* |  |  |
| Intercept | 11.15^***^ | 11.87^***^ |
| Talker Group |  |  |
| Stress |  |  |
| Task |  |  |
| Talker Group x Task |  |  |
| Talker Group x Stress |  |  |
| Stress x Task |  |  |
| Talker Group x Stress x Task |  |  |
|  |  |  |
| *Random Components* |  |  |
| Intercept | .56^***^ | .04^***^ |
| Residual | 1.48^***^ |  |
| Residual (IR, Attempt 1) |  | .09^***^ |
| Residual (IR, Attempt 2) |  | .07^***^ |
| Residual (IR, Attempt 3) |  | .07^***^ |
| Residual (IR, Attempt 4) |  | .05^**^ |
| Residual (STR, Attempt 1) |  | 4.56^***^ |
| Residual (STR, Attempt 2) |  | 4.90^***^ |
| Residual (STR, Attempt 3) |  | 4.92^***^ |
| Residual (STR, Attempt 4) |  | 4.97^**^ |
| ICC | .27 | IR [.31 to .44]  STR [.01 to .01] |
| AIC | 1422.34 | 1038.05 |

*Note.* IR: Immediate Repetition, STR: Short-Term Recall. ^*^ *p* < .05, ^**^ *p* < .01, ^***^ *p* < .001.

One may argue that low ICC values during short-term recall suggest that responses do not differ based on Attempt within Task and are, perhaps, unnecessary to the model (however, see Nezlek [1]). Regardless, due to the relatively *high* ICC values for immediate repetition, and because the analysis was designed to compare performance across tasks, it was necessary to account for task-specific order effects within participants to adequately compare group performance. Failure to do so may have misrepresented outcomes by not accounting for an individual participant’s proclivity to adjust their verbal accuracy during immediate repetition in response to auditory-orthographic cues.

1. Nezlek JB. An introduction to multilevel modeling for social and personality psychology. Social and Personality Psychology Compass. 2008; 2: 842-860. doi: 10.1111/j.1751-9004.2007.00059x
